# G-Exos: A wearable gait exoskeleton for walk assistance

**DOI:** 10.3389/fnbot.2022.939241

**Published:** 2022-11-10

**Authors:** Mouhamed Zorkot, Léa Ho Dac, Edgard Morya, Fabrício Lima Brasil

**Affiliations:** ^1^Neuroengineering Program, Edmond and Lily Safra International Institute of Neuroscience, Santos Dumont Institute, Macaiba, Brazil; ^2^Swiss Federal Institute of Technology, School of Life Sciences, Lausanne, Switzerland

**Keywords:** exoskeleton, foot drop, gait, assistive tecnology, human–machine interaction

## Abstract

Stroke is the second leading cause of death and one of the leading causes of disability in the world. According to the World Health Organization, 11 million people suffer a stroke yearly. The cost of the disease is exorbitant, and the most widely used treatment is conventional physiotherapy. Therefore, assistive technology emerges to optimize rehabilitation and functional capabilities, but cost, robustness, usability, and long-term results still restrict the technology selection. This work aimed to develop a low-cost ankle orthosis, the G-Exos, a wearable exoskeleton to increase motor capability by assisting dorsiflexion, plantarflexion, and ankle stability. A hybrid system provided near-natural gait movements using active, motor, and passive assistance, elastic band. The system was validated with 10 volunteers with foot drop: seven with stroke, two with incomplete spinal cord injury (SCI), and one with acute inflammatory transverse myelitis (ATM). The G-Exos showed assistive functionality for gait movement. A Friedman test showed a significant difference in dorsiflexion amplitude with the use of the G-Exos compared to gait without the use of the G-Exos [*x*^2^_(3)_ = 98.56, *p* < 0.001]. In addition, there was also a significant difference in ankle eversion and inversion comparing walking with and without the G-Exos [*x*^2^_(3)_ = 36.12, *p* < 0.001]. The G-Exos is a robust, lightweight, and flexible assistive technology device to detect the gait phase accurately and provide better human-machine interaction. G-Exos training improved capability to deal with gait disorders, usability, and motor and functional recovery. Wearable assistive technologies lead to a better quality of life and contribute using in activities of daily living.

## Introduction

Stroke is the second most common cause of mortality, the third leading cause of disability, and affects 15 million people worldwide yearly (Heidenreich et al., 2011). Stroke can occur either by an interruption of the blood flow (ischemic stroke) or by a blood vessel rupture (hemorrhagic stroke) and cause loss of muscle strength on one side of the body. Hemiplegia is a paralysis of one side of the body, whereas hemiparesis is a partial loss of movement of one side of the body (Setiawan et al., 2019).

**Figure 1:**
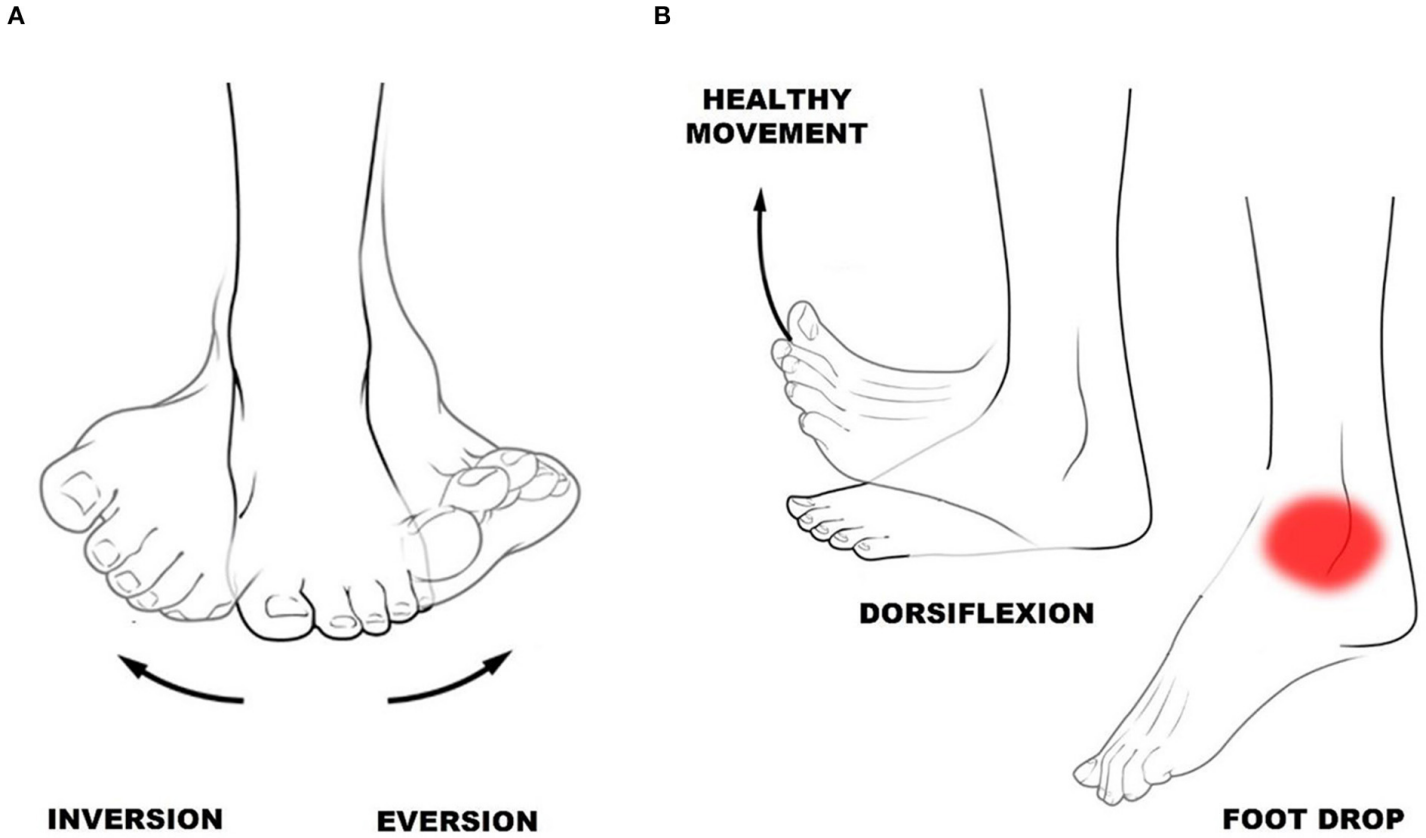
**(A)** Ankle inversion and eversion. **(B)** Ankle dorsiflexion—toes toward shins, and foot drop—difficulty lifting the forefoot.

The burden of stroke is exorbitant and distributed mainly in health care services, absenteeism, and drug treatments (Mozaffarian et al., 2015). Although physical therapy is essential to improve post-stroke outcomes, neurological and motor function recovery has limitations, and imposes caregiver dependency (Do et al., 2012). Generally, motor symptoms impair the gait, and foot drop is a walking challenge in which the ankle does not perform the dorsiflexion movement (Berenpas et al., 2019) (Figure 1). Since hemiparetic gait presents a reduction or absence of the heel strike subphase, the foot drop is overweighed (Isakov et al., 1992). Human gait presents sequential events to move. Gait can be divided into the support phase, when the foot is in contact with the ground and corresponds to 60% of the movement, and the swing phase, in which the limb remains in the air, corresponding to 40% of the movement (Perry and Bleck, 1993).

The ankle joint is one of the main ones responsible for the execution of the gait, presenting the function of transmitting to the lower limb the irregularities felt in the foot and developing an important function of the gait phase, the initial swing. This information helps maintain balance and the movements that can be performed are inversion, dorsiflexion, plantarflexion, adduction, and abduction (Araújo, 2010). Dorsiflexion and plantarflexion are essential movements to promote foot elevation and ankle inversion and eversion (Figure 1) for ankle stability.

Weakness and spasticity in the ankle of the hemiparetic limb contribute to the occurrence of foot drop in the swing phase of gait (Hou et al., 2019), occasioned by hypertonia and increased spasticity of the gastrocnemius muscle, in addition to the medial tibial and other muscles (Davids et al., 1992). Besides, there is an asymmetry in the eversion and inversion movements of the ankle. Thus, the gait is slower and more rigid with reduced cadence and step length, asymmetric, uncoordinated, and energy-consuming (Richards, 1995).

Post-stroke rehabilitation, based on the use of walkers, parallel bars, canes, weight support, orthoses, etc., is determinant for the reduction of motor sequelae. These approaches show significant results in rehabilitation, but still provide limited recovery of neurological and motor function, have long session duration, little engagement, and motivation (Awad et al., 2017). These therapies represent an opportunity for the development and insertion of novel assistive technologies (AT) and new therapeutic protocols for gait rehabilitation.

In the last decade, the development of robotic devices for gait assistance showed significant results, increasing the locomotion performance of post-stroke individuals (Veale and Xie, 2016; Awad et al., 2017; Quinlivan et al., 2017). For lower limbs, these devices are most commonly used to treat drop foot weakness and assist in ankle dorsiflexion movement, acting mainly in the support of gait's phase (Goldfarb et al., 2013). In recent years, wearable exoskeletons, the exosuits, gained prominence due to features such as lightweight, soft, and functional material (Awad et al., 2017). In addition, this technology can facilitate a more natural interaction between user and machine, reduce the interruption of the natural dynamics of walking (Veale and Xie, 2016), and reduce the energy cost during walking (Quinlivan et al., 2017).

As far as we are aware, the state-of-the-art device for rehabilitation of the foot drop is the wearable exoskeleton developed by Harvard University's engineering school for military and industrial applications. The ReStore^®^, from the company ReWalk^®^ - USA, is already available on the market. The system allows passive or active assistance of dorsiflexion and plantar flexion of the ankle, and its soft and lightweight material facilitates a natural gait pattern for hemiparetic individuals (Jasinski, 2020). The Biodesign Lab, at Harvard University, has also developed soft wearable robots that use innovative textile materials causing biologically appropriate movements, and passively assisting with specific tasks (Bae et al., 2017). These two models were used as a reference for the development of the G-Exos.

Functional electrical stimulation (FES), also used as an alternative to treat foot drop, presents interesting results and therapeutic effects, but is not yet consistent (Sannyasi, 2019). On the other hand, FES and ankle orthosis show better and more promising performance compared to conventional therapies (Moore et al., 2010). Some researches point out that FES presents advantages compared to ankle orthoses (AFOs), such as the lightness of the equipment, description, and instantaneous results only in dorsiflexion since the technology addresses only this movement. Despite that, the effects of FES diminish over time of use due to muscle fatigue. In contrast, AFOs have significant long-term advantages, allowing greater control in dorsiflexion, plantarflexion, application over a longer wearing time, motor learning, and neuroplasticity outcomes (Kemp, 2018; Sannyasi, 2019).

A critical challenge of scientific research has been the development of treatments that effectively provide rehabilitation and cost reduction to stroke patients. However, compared to research focused on upper limb rehabilitation of hemiparetic individuals, a scarcity of studies and projects targeting gait rehabilitation has been noticed in the literature. The most advanced existing technologies, such as FES or orthosis, have allowed the increase of patients' gait speed and length (Veale and Xie, 2016; Awad et al., 2017; Quinlivan et al., 2017), but still have limitations and show limited significant long-term results (Hobbs and Artemiadis, 2020). The reorganization of brain structure and neural networks in hemiparetic individuals, which results in improved long-term walking ability, is a gap that neuroscience is trying to address (Broccard et al., 2014). In addition, it is important to emphasize that most of the current devices do not allow the control and precise identification of the gait cycle.

In this context, we addressed the adaptation and usability related to better human–machine interaction (HMI) for assistive technology success, and effectiveness in long-term neurological and motor gait rehabilitation. Thus, the objective of this work was the development, validation, and functionality analysis of a wearable ankle exoskeleton, the G-Exos, for gait rehabilitation of individuals with foot drop. A user-centered technology and an accurate identification of the gait phase provide better human–machine interaction, clinical application, and assistive technologies success, which increases the quality of life, supports to rehabilitation professionals, and consequently, reduces public health costs.

## Materials and methods

### Quality function deployment

This project was approved by the Santos Dumont Institute ethics committee, CAAE: 41184020.4.0000.0129. After approval, focusing on the development of user-centered technology, online surveys were conducted with 20 individuals with stroke foot drop and 20 health professionals. The first questionnaire was the Stroke-specific Quality of Life Scale (Stroke-Scale), a standard health protocol submitted for analyzing the quality of life and limitations of individuals with stroke (Williams et al., 1999). The second and third questionnaires collected data from stroke individuals and health professionals, respectively, regarding the needs, restrictions, and requirements of using AT. These data were used for the development of the Quality Function Deployment - QFD Matrix - which is a tool that allows defining the premises of a project considering the user's opinion and the technical potentialities, which are known as customer requirements (CR) and quality characteristics (QC) (Akao and Mazur, 2003). The correlation between RC and CQ is done through a pattern of numerical codes, the number 1 given as a weak relationship between the parameters, 3 as a moderate relationship, and 9 as a strong relationship. The QFD matrix still relies on the quality house roof, allowing to perform the relationship between the QCs by making use of the symbols (– –) for very weak correlations, (–) for weak correlations, (+) for strong correlations, and (++) for very strong correlations. To complete the matrix, we performed market research and literature review on the state of the art of lower limb ATs and analyzed with the QCs considering a score between 1 and 5, in an increasing relation to the impact on the design characteristics. According to the results obtained from the interview, this same scale of relevance was considered for introducing the opinion of health professionals and individuals with foot drop in the matrix for the analysis and direction of the main premises that the project should take into account. Through the collected data, the G-Exos ankle brace was developed to meet the end user's needs besides the technical requirements.

### Development of the G-Exos

**Figure 2:**
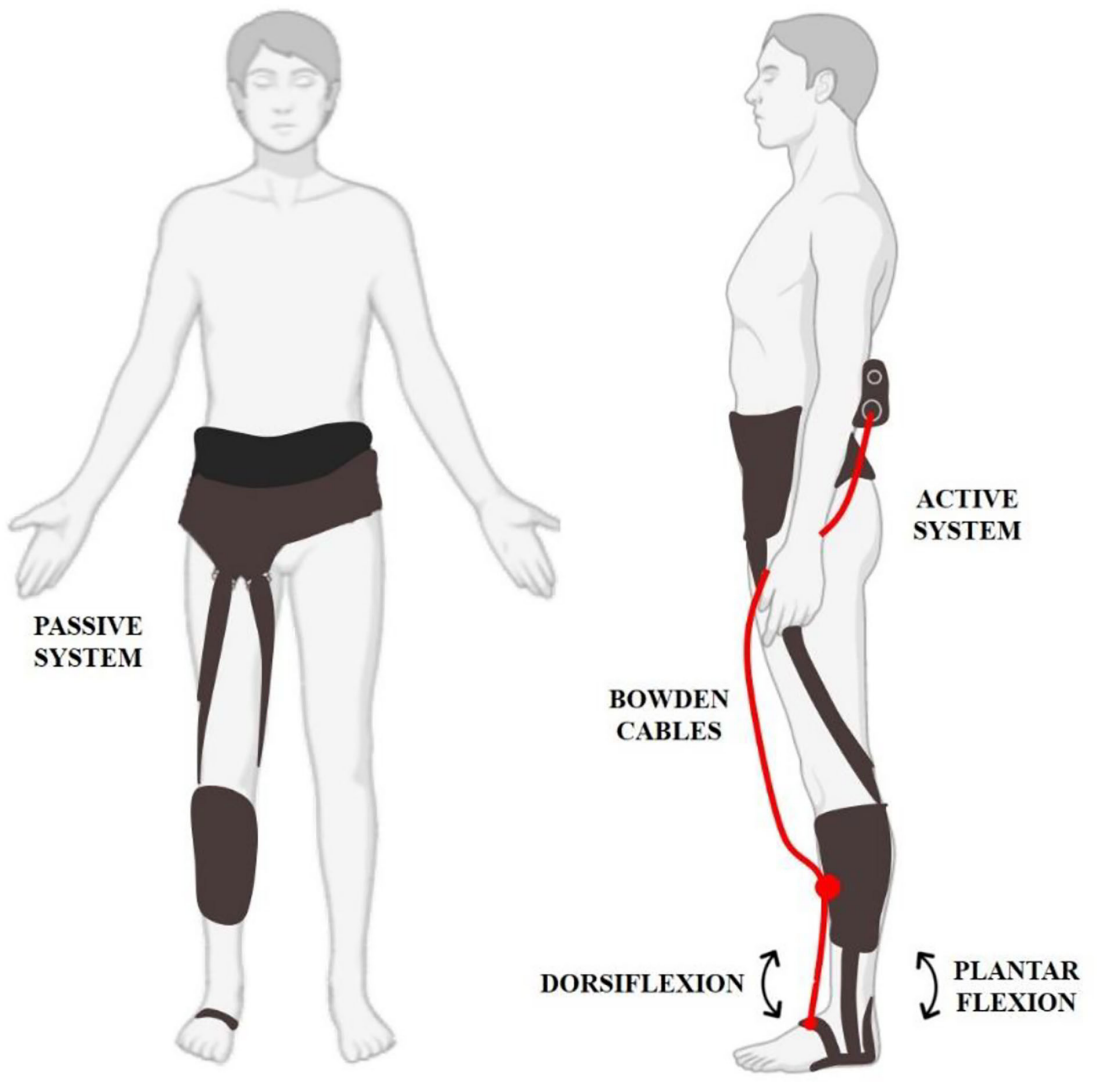
The G-Exos is a hybrid wearable exoskeleton composed of a passive system that uses the natural gait biomechanics to promote dorsiflexion assistance and ankle eversion/inversion through tensioned elastic bands. The active system uses an electromechanical system for greater assistance in ankle dorsiflexion movement. The hybrid system combines both systems, and allows the device to be configured according to the user's needs.

The G-Exos was developed considering a hybrid system: active, motor and hardware, and passive, elastic band, as given in Figure 2.

**Figure 3:**
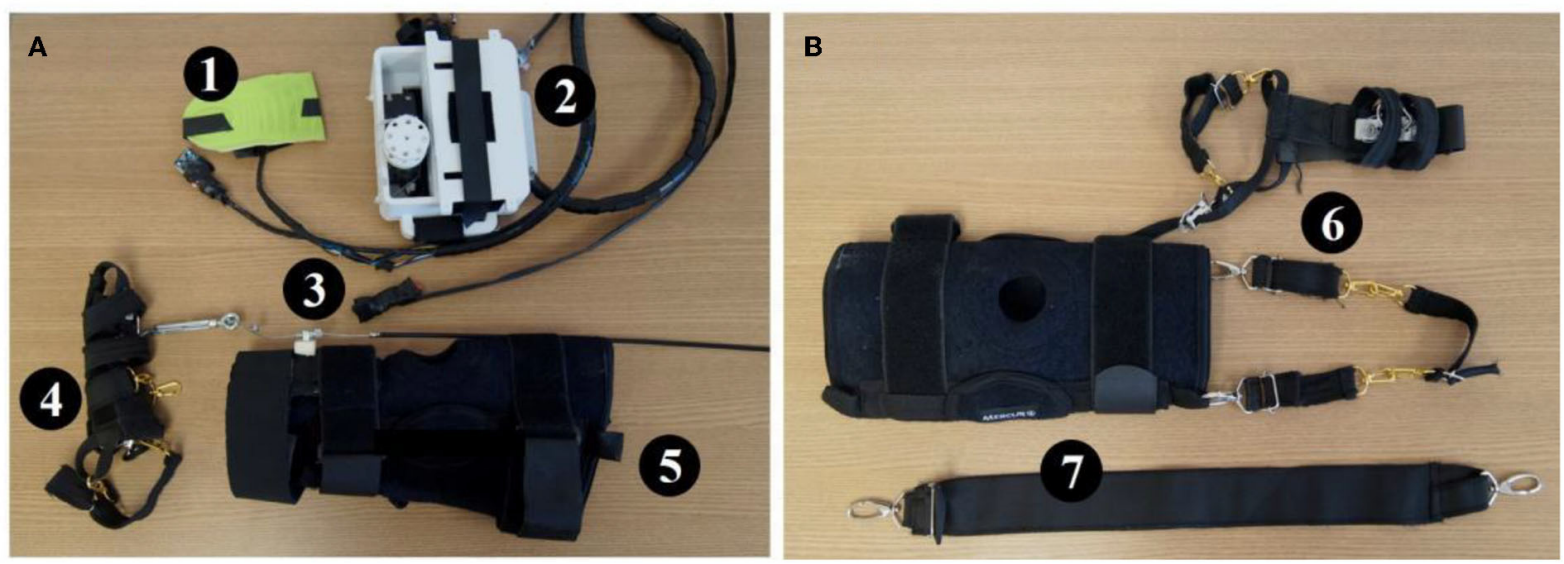
**(A)** Active system: (1) Insole with pressure sensor and sensor inertial. (2) Case to hold the servo motor, voltage regulator, battery, and microcontroller. (3) Emergency button. (4) Adapted firm foot strap. (5) Articulated knee brace with mechanism to hold a Bowden cable end connection. **(B)** Passive system: (6) Lateral elastic bandage. (7) Elastic knee bandage.

A tennis insole with embedded FSR402 resistive force sensors at the heel pad contact [Figure 3(1)], and a MPU6050 inertial measurement unit (IMU) at the heel counter were used to detect the gait phase [Figure 3(4)]. A Li-Ion battery (Tecnobattery, Brazil, 4.4Ah, 0.280 kg, 11.1V) powered the servo motor (Hitec HS-805 BB+ 180° 24.7 kg/cm High Torque Mega Quarter Scale) and a microcontroller (ATmega2560) enclosed in a 3D case to a waist belt [Figure 3(2)]. The open-source Arduino integrated development environment software (Arduino IDE) was used to generate the control code system.

The mechanical system consisted of an articulated neoprene knee-pad (Mercur, Brazil) to stick firmly the spiral end connection of a Bowden cable and transmit mechanical force from the servo-motor to pull the instep (ankle dorsiflexion).

The passive system refers to adjustable elastic bandages made with carabiners to generate binary sets of support as a function of the gait cycle (Awad et al., 2017). In addition, these bandages were also used to assist in controlling ankle eversion and inversion and consequently promote ankle stability.

### Identifying gait phases

**Figure 4:**
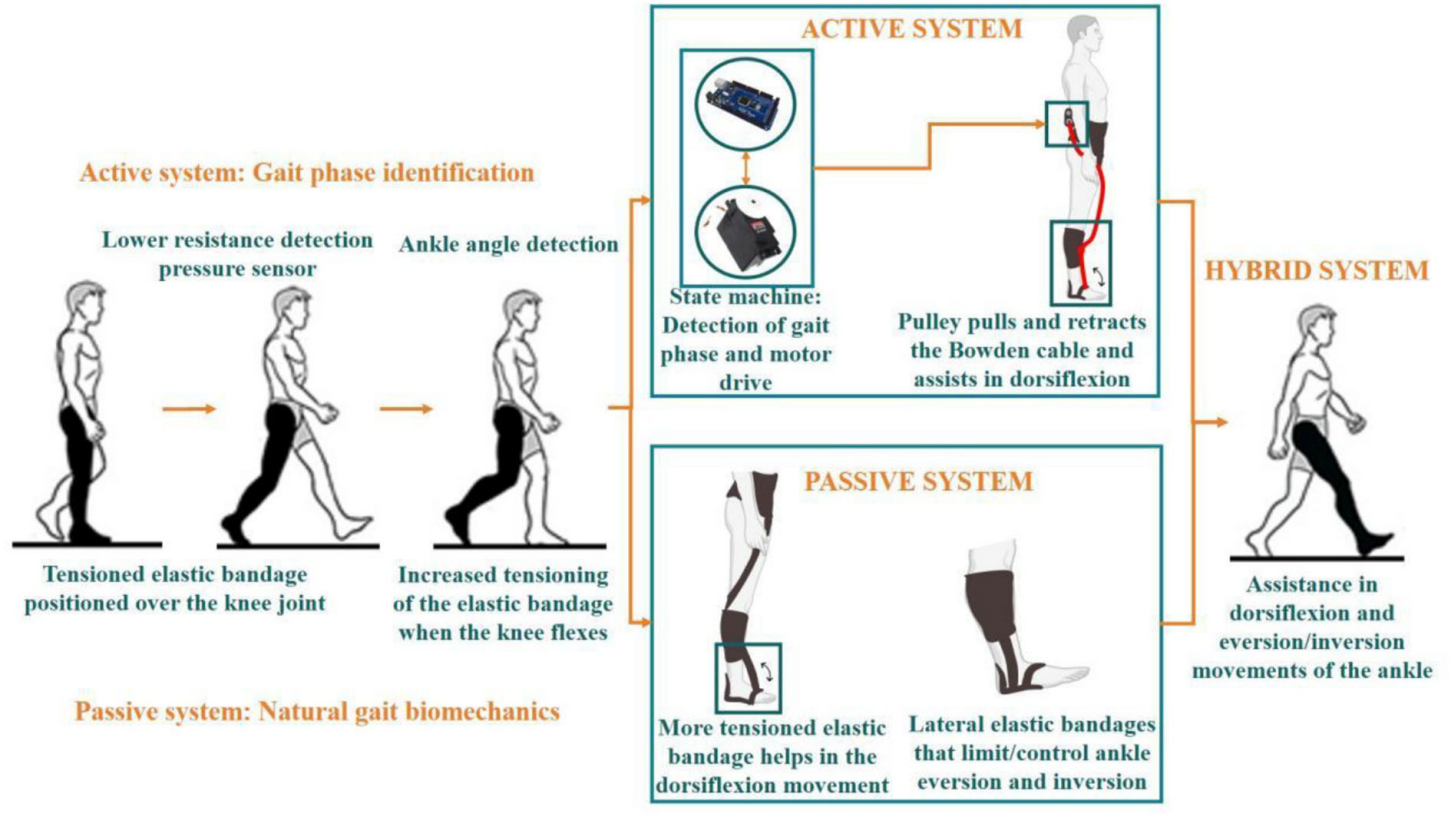
G-Exos can be configured in three modes: active system, passive system, and hybrid (active + passive). The principle of operation is represented in the figure. **Active system operation:** has embedded hardware, composed of two FSR's and an IMU, for the identification of the transition from the contact phase to the swing phase of the gait. With the integration of the sensor operation with the state machine method, the first condition of the firmware is to identify the decrease of resistance on the ankle, and then the condition of the ankle angulation range. When these conditions are met, the microcontroller triggers the servo motor which, when spinning together with a pulley, converts electrical energy into mechanical energy, pulling the Bowden cable and, consequently, helping to perform the ankle dorsiflexion. **Passive system operation:** Making use of the natural gait biomechanics itself, with tensioned elastic bands strategically positioned at the knee joint and one extremity at the tip of the foot, when the user flexes the knee, the elastic band is more tensioned and, consequently, assists in the execution of the dorsiflexion movement. Furthermore, with the lateral elastic bands positioned parallel to the ankle, their tension can be adjusted to control the amplitude of ankle eversion and inversion movement. **Hybrid system operation:** association of the active system with the passive system. Video link: https://www.youtube.com/watch?v=dAwfYoUrbgM.

Human gait repetitive pattern allows gait phase detection according to the foot–ground contact, which is generally divided into support phase and swing phase. Gait research and analysis generally use vertical ground reaction force (vGRF) as the best parameter to extract characteristics of gait phases (Lim et al., 2020). The control of the G-Exos was performed by analyzing the vGRF recorded from FSRs under the heel pad, and additionally the inertial sensor to improve the gait phase identification. The developed firmware was composed of the state machine, in which an order of execution is imposed at a time to direct the proposed operation. In other words, with the data collected from the FSRs and the IMU sensor, heel pressure conditions and ankle angulation were imposed to identify pre-balance phase of the gait, a prior phase to the initial swing, that is, the moment when the G-Exos should promote assistance for ankle dorsiflexion (Kim et al., 2005). The operating principle of the system is given in Figure 4.

### Experiment

For safety reasons, the experiment was initially performed at the Edmond and Lily Safra International Institute for Neuroscience (ELS-IIN) with four healthy volunteers to test the performance and analyze the functionality of the G-Exos. These volunteers walked twice (round trip) a pre-defined distance of 5 m for four conditions: without the G-Exos, with the G-Exos hybrid mode, with G-Exos active mode, and with G-Exos passive mode.

Ten volunteers with foot drop underwent the experiment (Table 1), seven with hemiparetic stroke, two with incomplete spinal cord injury (SCI), and one with acute inflammatory transverse myelitis (ATM). Motor function was measured by the muscle strength grading scale (Oxford Scale), which is a method used in physical therapy for the assessment and classification of muscle strength. The tool has a measurement scale of 0 to 5, and is described as 0, No contraction; 1, Visible/palpable muscle contraction but no movement; 2, Movement with gravity eliminated; 3, Movement against gravity only; 4, Movement against gravity with some resistance; 5, Movement against gravity with full resistance (Clarkson, 2000). It is worth mentioning that the G-Exos at this moment was developed for hemiparetic individuals, since it promotes the assistance of one limb. However, as an exploratory work, the experiment was also performed with volunteers with SCI, who present a foot drop in both legs and with ATM. All volunteers performed the walk on the ground without the use of the G-Exos, and with the G-Exos configured in three modes: hybrid system, active system, and passive system. For each configuration, the volunteers performed three laps (round trip) of a pre-defined distance of 3.5 m. A rest between trials was considered according to the limitation of the volunteers with a foot drop. To validate the functionality of the G-Exos, the experiment was tested in only one session with each volunteer. In a future study, we aim at clinical validation by conducting a larger number of sessions and follow-up for a longer period.

**Table 1:** Characteristics of volunteers with foot drop.

	**Age**	**Injury**	**Muscular strength grading scale**	**Gender**		**Leg with G-Exos**		**Injury time (years)**
				**Male**	**Female**	**Right**	**Left**	
**N1**	46	Stroke	3		x	x		7
**N2**	47	Stroke	3	x			x	4
**N3**	52	Stroke	4	x			x	8
**N4**	62	Stroke	2		x		x	9
**N5**	63	Stroke	3		x		x	19
**N6**	32	Stroke	2		x	x		5
**N7**	62	Stroke	3	x		x		6
**N8**	56	SCI	4	x		x		3
**N9**	23	SCI	2	x			x	9 months
**N10**	57	ATM	3		x		x	8

Classification muscular strength grading scale, the Oxford Scale. The tool has a measurement scale of 0-5, and is described as 0, No contraction; 1, Visible/palpable muscle contraction but no movement; 2, Movement with gravity eliminated; 3, Movement against gravity only; 4, Movement against gravity with some resistance; 5, Movement against gravity with full resistance.

To prevent any falls and to analyze the performance overground, volunteers with foot drop walked using ZeroG^®^, a device for gait training in safe (Hidler et al., 2011). However, the volunteers were not suspended at any moment, using the device strictly to avoid the risk of falling. The total time of the experiment was ~1.30 h per volunteer.

To perform the protocol of the proposed experiment, carried out using the hybrid system (active + passive), the active, the passive, and without the use of G-Exos, a wireless sensor Delsys Trigno (Delsys, USA), also composed of an IMU sensor, was inserted in the dorsum of the foot to capture ankle angulation. Data from these sensors were recorded in EMGworks^®^ software (Delsys, USA).

### Data processing and statistical analysis

Inertial measurement unit data were analyzed in custom Matlab^®^ scripts (Mathworks, USA). Contact phase and swing phase of the gait were extracted from the videos and synchronized with IMU data according to the execution time of the gait. A low-pass filter of the third order, 12 Hz was applied to keep the data related to gait phase movement (Verplaetse, 1996; Chang et al., 2016), and a 0.5 Hz high-pass filter was applied to adjust the drift.

For the statistical analysis, a vector was created with the maximum amplitude of dorsiflexion and ankle inversion/eversion of each step performed. The statistical analysis was done with SPSS^®^ software 26.0 (IBM, USA). To validate the functionality of the G-Exos with the data collected with the use of the hybrid system, the active, the passive and without the use of the G-Exos, the following parameters were analyzed and compared: gait events, ankle angulation for the dorsiflexion movement, and ankle angulation for the eversion and inversion movements.

To check if the G-Exos promotes assistance in the dorsiflexion movements and control in the ankle eversion and inversion movements, a statistical analysis was performed for both data collected to verify whether there was a significant difference in the amplitude of movements with the use of the G-Exos, in the three configurations (hybrid system, active, and passive), when compared to walking without the use of the G-Exos.

Some groups presented a normal distribution and others a non-normal distribution and, aiming at a more conservative analysis and due to the low number of volunteers, a non-normal distribution was considered for all cases and, consequently a non-parametric analysis, which presents more conservative conclusions. The analysis was done for dorsiflexion, eversion, and inversion movements of the ankle, both on the ground, with the volunteers with the foot drop.

Initially, a Friedman test was performed to verify whether there was a significant difference between the amplitude of ankle dorsiflexion and eversion/inversion movements without the use of the G-Exos and with the use of the G-Exos configured with the hybrid system, active and passive. Then, after verifying the significant difference in all cases, *post-hoc* analysis was performed with the Wilcoxon Test for the unpaired comparison of two groups, between walking without the use of the G-Exos with each of the configurations mentioned (hybrid system, active and passive) and also the pairwise comparison between the systems with the use of the G-Exos.

In addition, a complementary analysis compared the systems vs. users of the accuracy of identification of the gait phase, and speed and time to perform the gait in the predefined path.

## Results

### QFD matrix

Surveys data of hemiparetic individuals and health professionals entered a QFD matrix, considering the volunteers' answers and transforming them into customer requirements. The matrix was also filled out comparing the functionalities of the main technologies and state-of-the-art in gait assistance for hemiparetic individuals, according to the customer requirements. The degree of priority, collected in the interviews, was also filled in.

**Figure 5:**
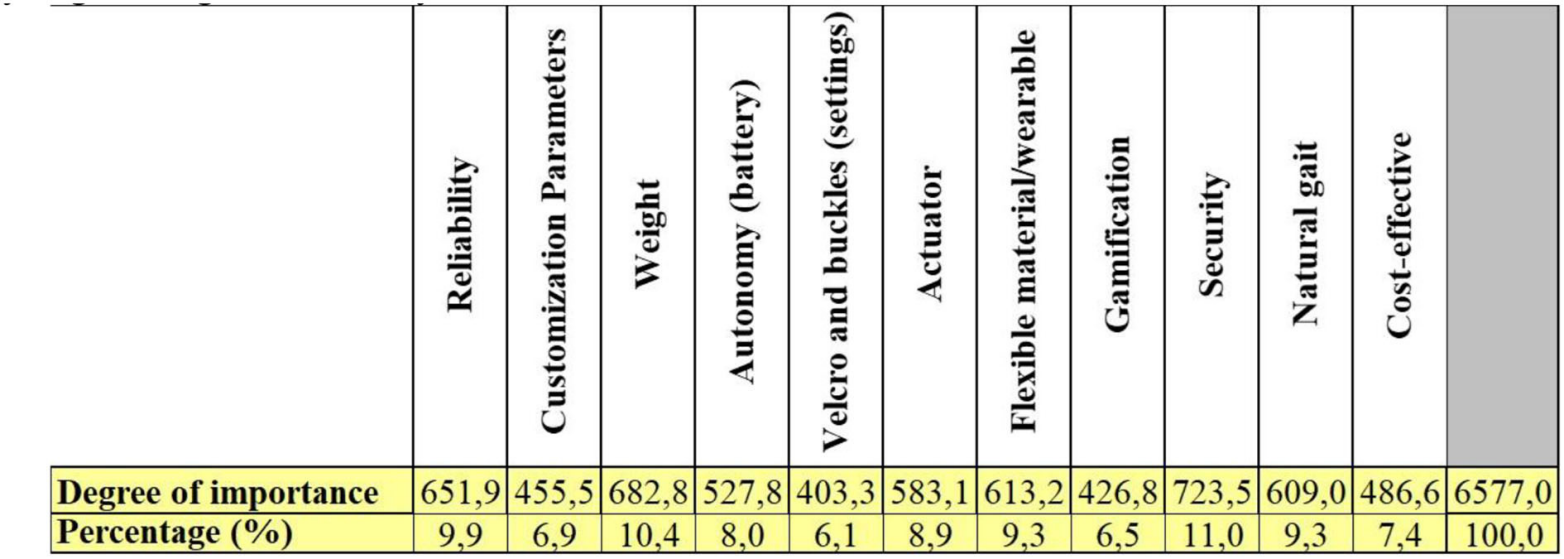
Main features that the G-Exos should meet obtained according to the results of the QFD matrix.

As shown in Figure 5, it was possible to define the main features that the G-Exos should meet, considering the user-centered technology being, in descending order: safety, lightweight, reliability, and flexible material.

### Prototype

**Figure 6:**
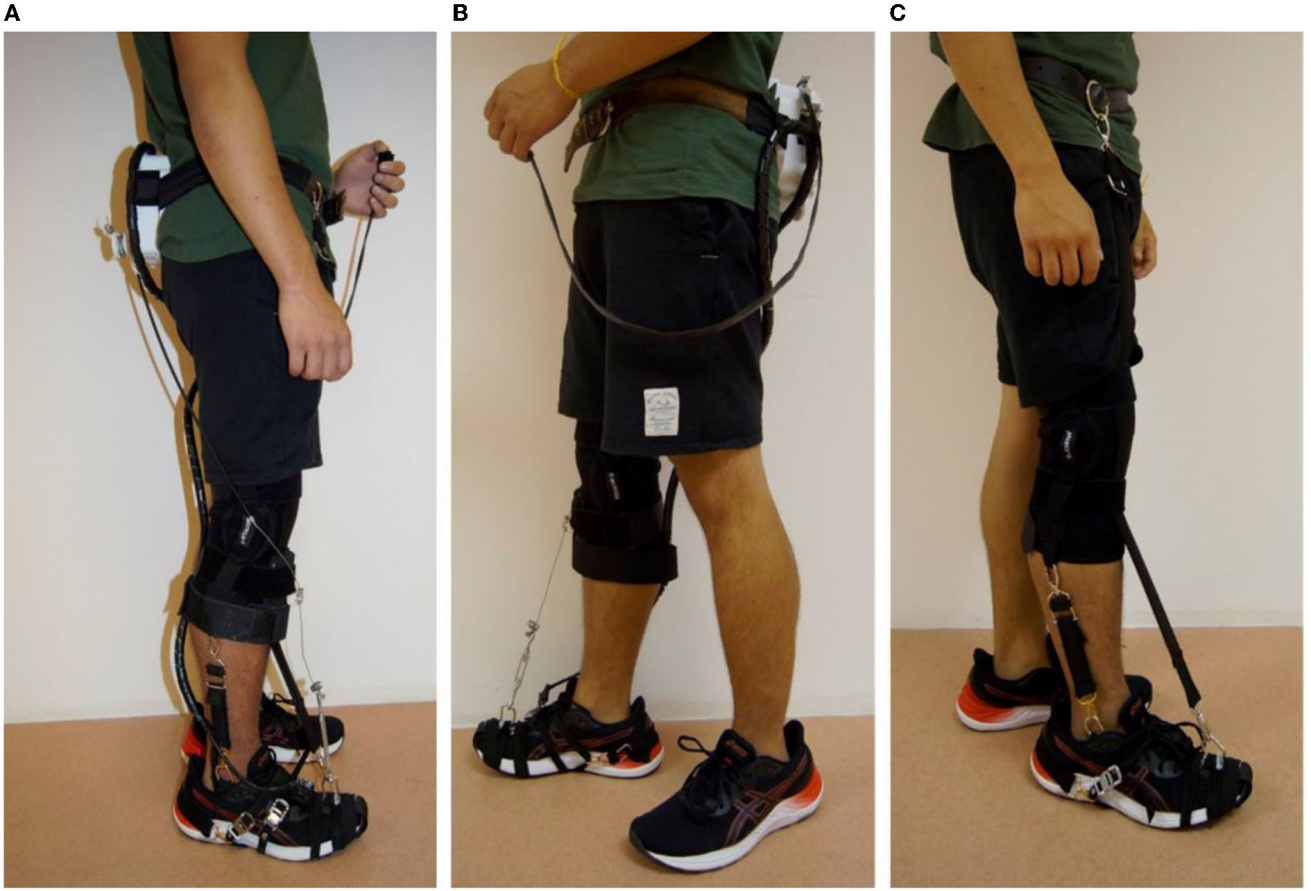
G-exos prototype. **(A)** Hybrid system. **(B)** Active system. **(C)** Passive system.

The G-Exos can be used in three configurations: hybrid, active or passive. The hybrid system associates the mechanism of the active system with the passive system with greater gait assistance through the motor, and ankle inversion/eversion stability through the passive system, in addition to assisting the ankle's dorsiflexion movement. The system configuration is shown in Figure 6A.

When the G-Exos is configured for the active system only (Figure 6B), it is possible to promote significant assistance in the ankle dorsiflexion movement through the built-in motor, mechanism, and hardware. In contrast, the mode does not allow assistance for the ankle eversion and inversion movements. Finally, the passive system (Figure 6C) has two elastic bandages in parallel with the ankle and are attached by carabiners to the fixed foot strap to promote assistance in controlling the eversion and inversion movements and, consequently, ankle stability. This system also has an elastic bandage that assists the dorsiflexion movement through the natural biomechanics of walking.

The hybrid system weighs 1.88 kg, while the active system weighs 1.74 kg and the passive system weighs 0.48 kg. The battery life, in the hybrid and active systems, is ~3 h. The approximate cost to build the prototype was $330.

### G-Exos accuracy in identifying gait phase

#### Analysis of the detection of walking events using the recorded videos

Identifying the gait phase accurately is a determining factor for control in active systems and better HMI. This was analyzed according to the number of steps detected, i.e., if the motor was activated at each step performed with the leg. Table 2 shows the results of the gait identification performed by the G-Exos onboard system for the hybrid system and the active system, both on the ground l.

**Table 2:** Accuracy of G-Exos in identifying gait in volunteers with a foot drop.

	**Volunteers with foot drop**												
	**N1**			**N2**			**N3**			**N4**		
	**Steps**		**Accuracy**	**Steps**		**Accuracy**	**Steps**		**Accuracy**	**Steps**		**Accuracy**
	**Taken**	**Identified**		**Taken**	**Identified**		**Taken**	**Identified**		**Taken**	**Identified**	
Hybrid	50	50	100.00%	81	77	95.06%	50	50	100.00%	42	35	83.33%
Active	51	51	100.00%	47	42	89.36%	50	50	100.00%	46	42	91.30%
Accuracy per volunteer	100.00%			92.21%			100.00%			87.32%		
	**N5**			**N8**			**N9**			**N10**		
	**Steps**		**Accuracy**	**Steps**		**Accuracy**	**Steps**		**Accuracy**	**Steps**		**Accuracy**
	**Taken**	**Identified**		**Taken**	**Identified**		**Taken**	**Identified**		**Taken**	**Identified**	
Hybrid	56	48	85.71%	40	39	97.50%	23	15	65.22%	33	33	100.00%
Active	47	30	63.83%	41	40	97.56%	18	17	94.44%	29	29	100.00%
Accuracy per volunteer	74.77%			97.53%			79.83%			100.00%		
Overall accuracy	94.87%											

An initial configuration was used for each volunteer to obtain a better performance of the G-Exos in identifying the gait phase and, consequently, higher HMI. The percentage of accuracy of the control and hardware system embedded in the G-Exos to identify the gait phases can be analyzed in a generalized way for the volunteers with foot drop and individually. Due to physical limitations, volunteers N6 and N7 did not wear the G-Exos configured in the hybrid active system and, for this analysis, only the volunteers who made use of the hybrid and active system were considered.

It is possible to observe a mean overall accuracy higher than 94% in the steps identified by the G-Exos.

#### Analysis of gait event detection by the IMU sensor

The IMU data were processed to obtain the ankle motion behavior according to the gait phases. These data were used for statistical analysis, which will be seen in the next section. To obtain the amplitude of the dorsiflexion movement, the moment when the foot of each volunteer was in the neutral position, foot without inclination, was adjusted and the difference was calculated with the maximum point of movement amplitude for each step (Chang et al., 2016).

**Figure 7:**
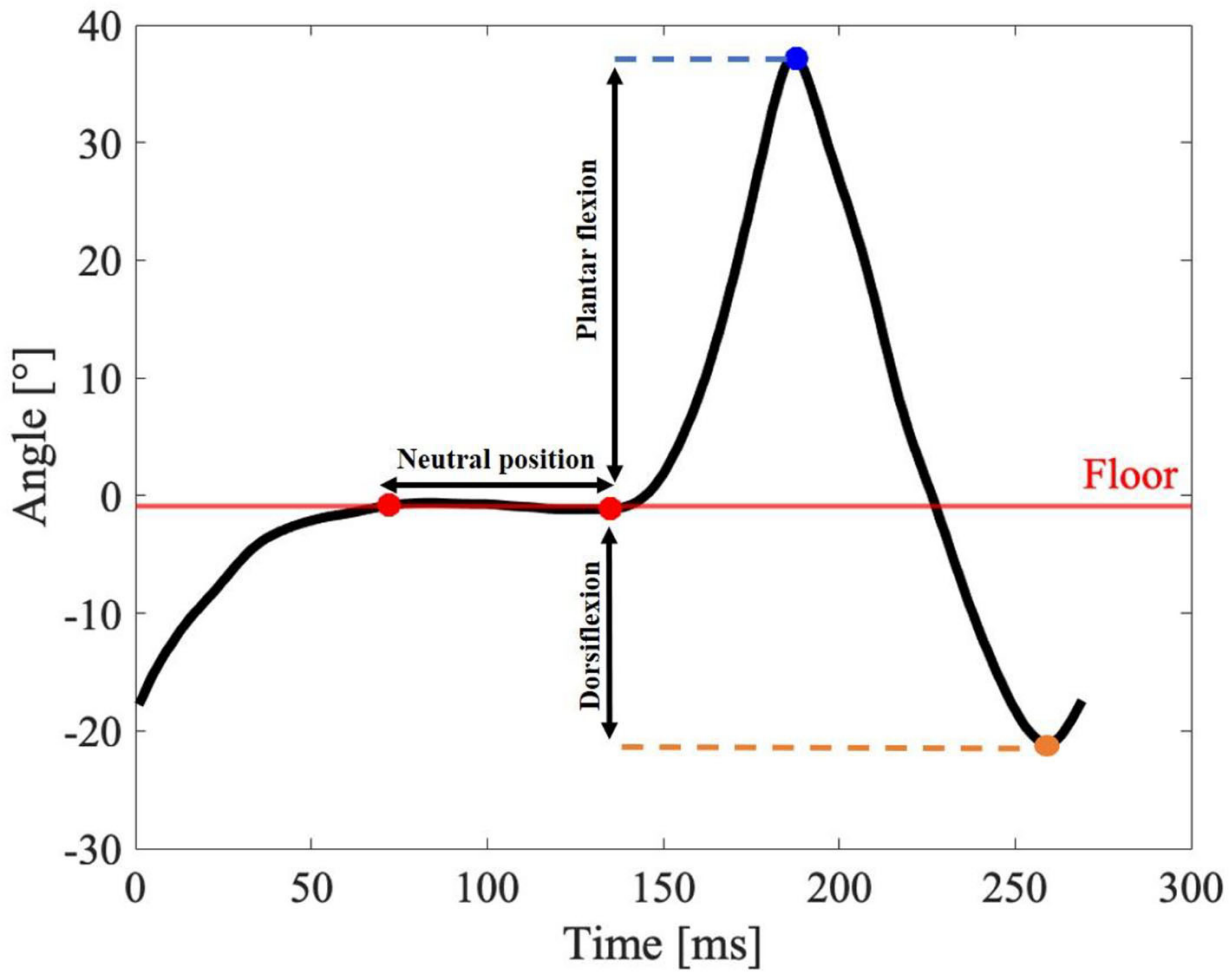
Illustrative ankle range of motion of volunteer N1. Obtaining the amplitude of the dorsiflexion movement during the execution of a step, with detection of the neutral position demarcated by the red dots and the maximum amplitude of the movement demarcated by the blue dot. The green dot refers to the plantar flexion movement.

In Figure 7, it is possible to observe the amplitude of the dorsiflexion movement when performing a step, with the detection of the neutral position demarcated by the red dots and the maximum amplitude of the movement demarcated by the blue dot. The green dot refers to the plantar flexion movement. Since the volunteers have the foot drop, the greatest interest is in obtaining the amplitude of the dorsiflexion movement.

**Figure 8:**
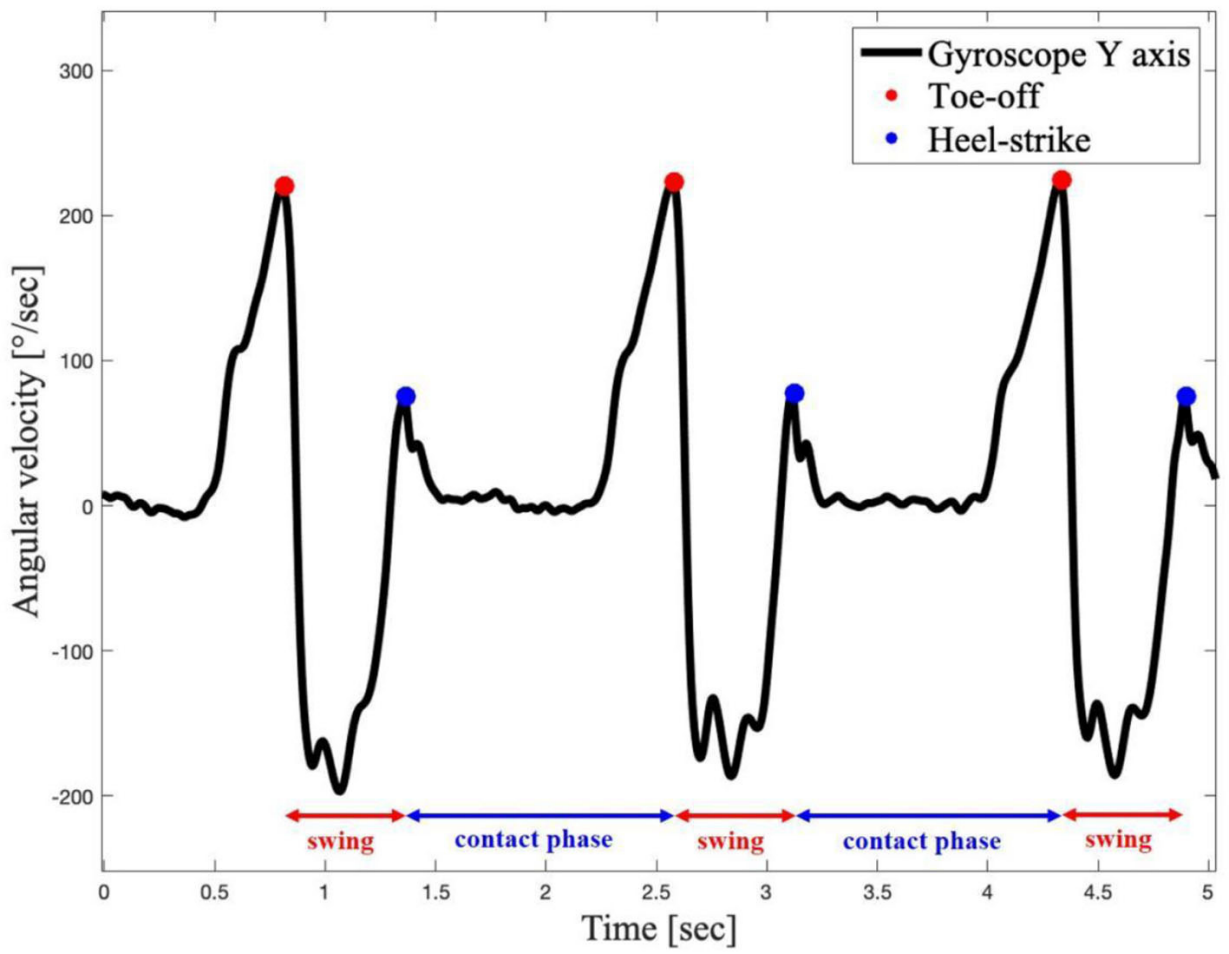
Gait events of volunteer N1 performing three steps.

The gait can be divided into a contact phase, when the foot is in contact with the ground, and a swing phase, when the foot is not in contact with the ground. This is the phase in which the dorsiflexion movement should be assisted. Figure 8 shows the gait events of a volunteer performing three steps with the G-Exos support.

Figure 8 presents the contact and swing phases of the gait, and the final contact and the initial contact of the foot with the ground. The transition between these two stages, respectively, is the instant when the G-Exos detects, with the onboard hardware system, the moment when the dorsiflexion movement should occur and, consequently, be assisted by the device in the active system.

### Functionality and usability

**Figure 9:**
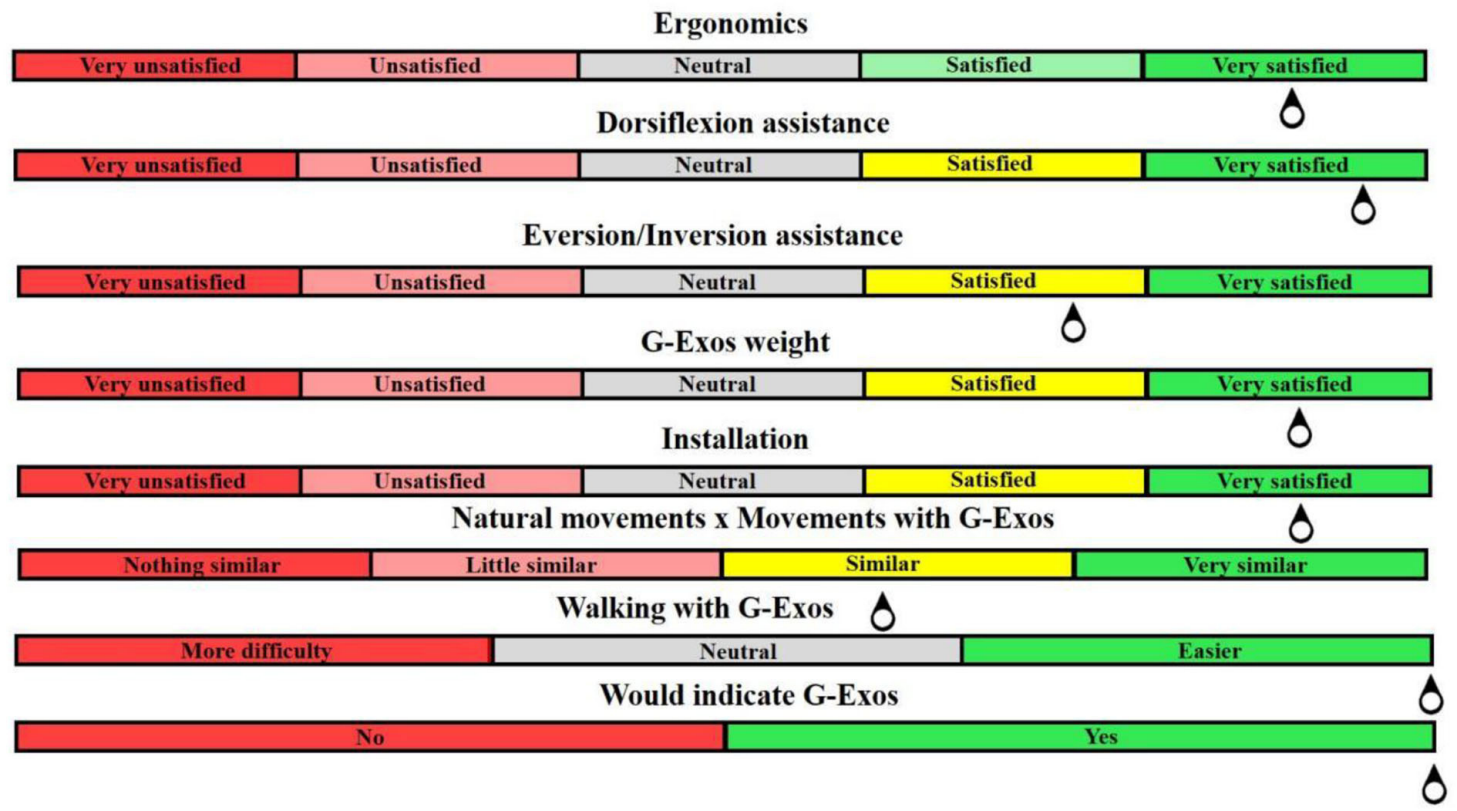
Summary of the results obtained from the questionnaires applied to the functionality of G-Exos.

The user experience with the G-Exos technology and the protocol were analyzed from the feedback questionnaire. Ten volunteers mentioned the greater ease in performing the ankle dorsiflexion movement during gait, promoted by the G-Exos assistance in the hybrid system, active and passive. Therefore, according to the volunteers' analysis, the three systems proved to be functional in assisting the dorsiflexion movement. The ankle stability by reducing the amplitude of eversion and inversion movement was possible through the passive system and, consequently, the hybrid system. This was noticed in the volunteers with foot drop. A summary of the results obtained through the questionnaire applied to the volunteers is shown in Figure 9.

Regarding the usability of the system, 10 volunteers with foot drop were very satisfied with the weight and ergonomics of the equipment, for being a flexible system, and satisfied with the installation of the system. When asked if the assisted movements are similar to the natural movements of the leg, seven hemiparetic volunteers and the ATM volunteers considered it very similar, while two volunteers with SCI considered the movement not very similar to the natural one.

### Gait speed

With the hypothesis that the assistance of dorsiflexion, eversion, and inversion movements performed by the G-Exos promotes a greater speed to perform the gait, an observational analysis was performed on the speed of each volunteer with foot drop with the use of the system, configured in hybrid, active and passive mode, before and after the use of the G-Exos. As already mentioned, due to physical limitations, volunteers N6 and N7 did not use the hybrid system and the active system. According to the timed interval and the predetermined distance, the estimated speed of the gait in the execution of the experiment was obtained, as shown in Table 3.

**Table 3:** Speed in m/s when walking.

	**Volunteers with foot drop**			
	**N1**	**N2**	**N3**	**N4**	**N5**
Before G-Exos	0.35	–	0.36	–	0.27
Hybrid	0.39	0.18	0.39	0.18	0.29
Active	0.38	0.26	0.41	0.21	0.33
Passive	0.39	0.37	0.35	0.18	0.31
After G-Exos	0.47	0.37	0.37	0.25	0.30
	**N6**	**N7**	**N8**	**N9**	**N10**
Before G-Exos	0.13	–	0.61	0.08	–
Hybrid	–	–	0.63	0.15	0.31
Active	–	–	0.62	0.14	0.32
Passive	0.17	0.19	0.70	0.10	0.31
After G-Exos	0.15	0.37	–	–	0.35

Due to the effort and physical limitations, some of the volunteers experimented without the G-Exos only before or after, which will be analyzed in the discussion.

### Statistical analysis

For the statistical analysis of foot drop, six volunteers were considered because other four volunteers presented significant spasticity and inversion/eversion of the ankle, which interfered with the reference coordinates of the IMU sensor and, consequently, with the quality of the data signals collected. The data used for the statistical analysis were from volunteers N1, N2, N3, N4, N8, and N10.

In Table 4, it is possible to verify the median amplitude of movement for the foot drop group for both dorsiflexion (Table 4a) and eversion/inversion movements (Table 4b) without the G-Exos and with the use of G-Exos. When compared to the range of motion without the use of the G-Exos, it is possible to verify that the G-Exos promoted an increase in the amplitude of the ankle dorsiflexion movement in the hybrid, active and passive system (Table 4a) for the users with foot drop and, consequently, indicating the better performance of the movement with the assistance promoted by the G-Exos. Furthermore, in Table 4b, it is possible to verify that the G-Exos promoted a reduction in the amplitude of the ankle eversion/inversion movement in the hybrid, active, and passive system for the users with foot drop, indicating that the use of the G-Exos aided in the stability of the ankle of the user.

**Table 4:** Comparison of ankle range of motion with and without the use of the G-Exos for the foot drop group.

**(a) Amplitude of ankle dorsiflexion movement**			
	**Without G-Exos**	**Passive**	**Active**	**Hybrid**
Median (cm)	5.79	14.60	15.60	18.30
**(b) Amplitude of ankle eversion/inversion movement**				
	**Without G-Exos**	**Passive**	**Active**	**Hybrid**
Median (cm)	18.30	11.50	11.60	8.49

For partial gait assistance, it is expected that G-Exos will promote increased dorsiflexion range of motion (a) and reduced ankle eversion/inversion range of motion (b).

#### Range of motion of ankle dorsiflexion

**Figure 10:**
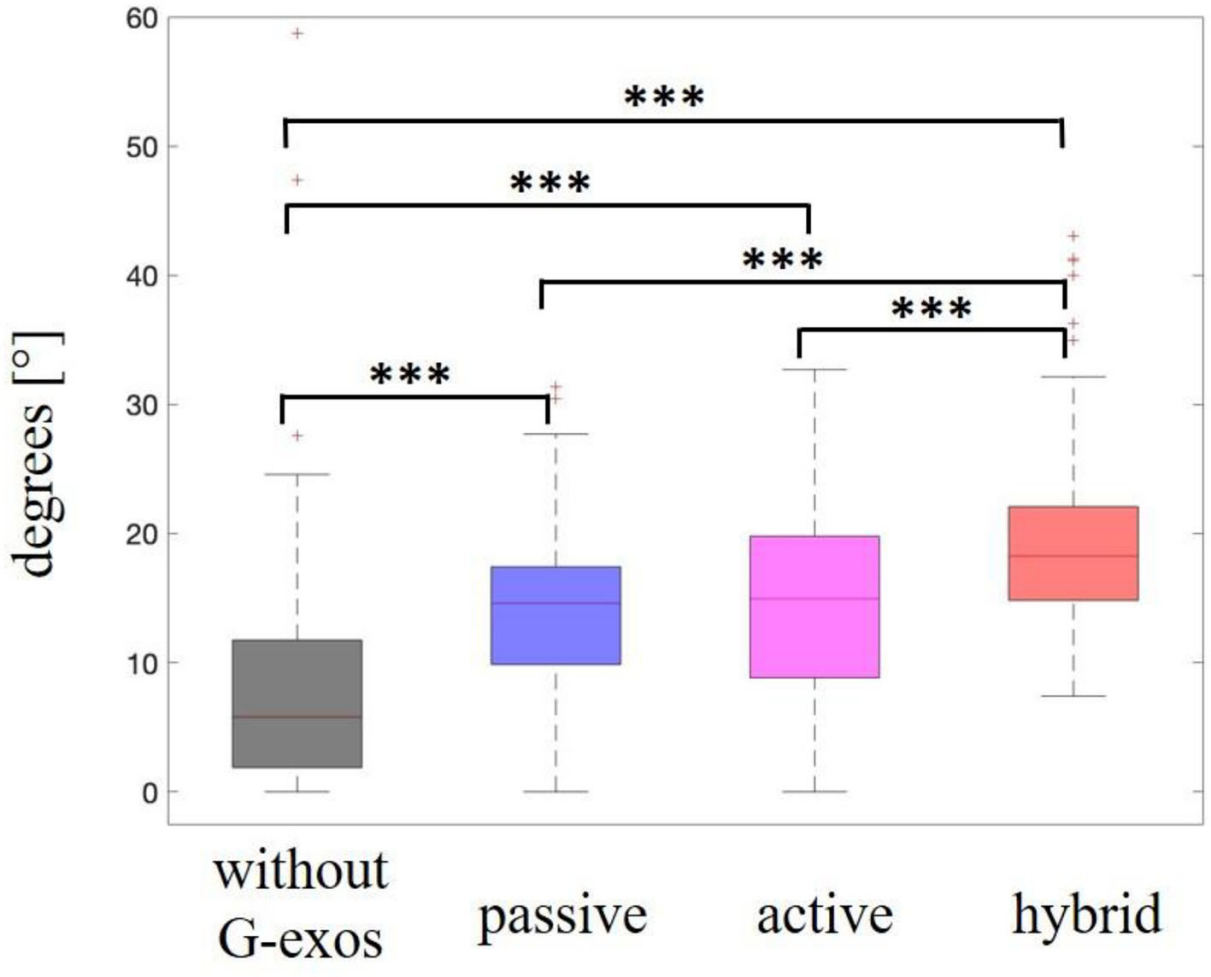
Range of motion of ankle dorsiflexion in volunteers with foot drop. Statistically significant differences are shown with the stars (***p-value < 0.001). Here we see a comparison of range of motion of ankle dorsiflexion without the use of the G-Exos, with the passive system, with the active system, and with the hybrid system (active and passive), and only the comparison of the passive and active systems was not significantly different. Median represented in the second quartile.

A Friedman test showed that there was a significant difference between the dorsiflexion range of motion scores for the dropped foot group with the use of the G-Exos vs. natural walking without the use of the G-Exos on the ground [*x*^2^_(3)_ = 98.6, *p* < 0.001]. A *post-hoc* test using a Wilcoxon's *t*-test indicated that for the amplitude of dorsiflexion movement, there is a significant difference between the hybrid G-Exos system (Mdn = 18.3) and the gait without G-Exos (Mdn = 5.79) on the ground, *Z* = 9.07, *p* < 0.001; between the active G-Exos system (Mdn = 15.0) and gait without G-Exos (Mdn = 5.79), *Z* = 6.38, *p* < 0.001 and between the passive G-Exos system (Mdn = 14.6) and l gait without G-Exos (Mdn = 5.79), *Z* = 7.49, *p* < 0.001. All statistical results are given in Figure 10.

#### Range of motion of ankle inversion and eversion

**Figure 11:**
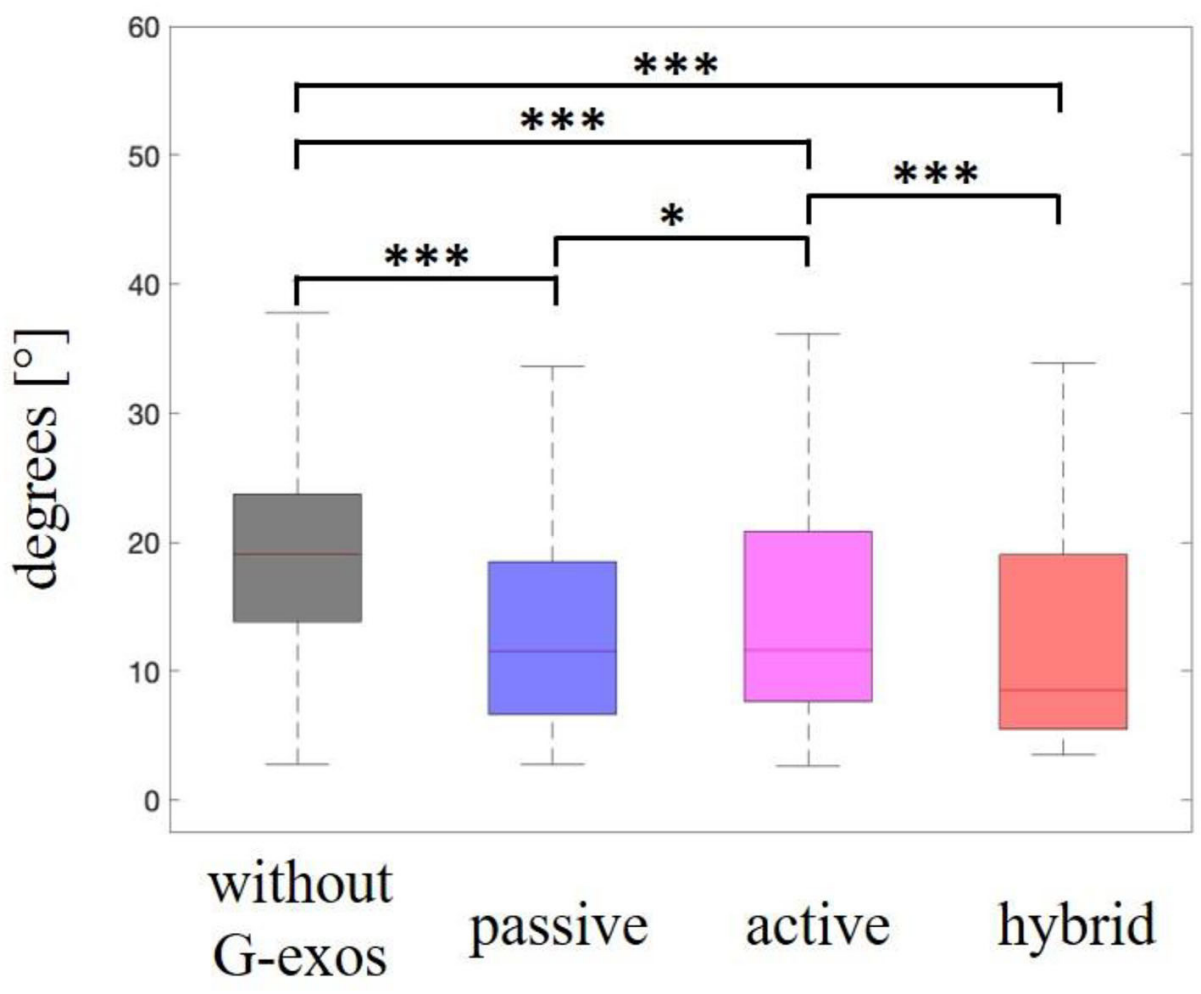
Range of motion of ankle inversion/eversion in patients with a foot drop. Statistically significant differences are shown with the stars (*p-value < 0.05, ***p-value < 0.001). Here, we see a comparison of range of motion of ankle inversion/eversion without the use of the G-Exos, with the passive system, with the active system, and with the hybrid system (active and passive). Median represented in the second quartile.

Friedman's test showed that there was a significant difference between the ankle eversion and inversion range of motion scores for the foot drop group with the use of G-Exos vs. natural walking without the use of G-Exos on the ground (*x*^2^_(3)_ = 36.1, *p* < 0.001). A *post-hoc* test using a Wilcoxon's *t*-test indicated that for the amplitude of ankle eversion and inversion movement, there is a significant difference between the G-Exos hybrid system (Mdn = 8.49) and the gait without G-Exos (Mdn = 18.3) on the ground, *Z* = 5.99, *p* < 0.001; between the active G-Exos system (Mdn = 11.6) and gait without G-Exos (Mdn = 18.3), *Z* = 3.24, *p* = 0.001 and between the passive system of G-Exos (Mdn = 11.5) and the gait without G-Exos (Mdn = 18.3), *Z* = 3.70, *p* < 0.001. As expected, there was no significant difference between the G-Exos active system (Mdn = 11.6) and the natural gait without G-Exos (Mdn = 18.3), *Z* = 5.47, *p* < 0.001. All statistical results are shown in Figure 11.

## Discussion

Stroke is one of the most common causes of motor disability in the world, affecting thousands of people and increasing public health costs (Mozaffarian et al., 2015). Current therapies show significant results but are limited to the level of motor and neurological rehabilitation. In this context, the aim of this work was the development and validation of a wearable ankle exoskeleton to promote gait assistance helping in dorsiflexion, eversion, and inversion movements of stroke victims, or other conditions who present walking difficulties.

To promote greater adoption, usability, and safety in the use of an exoskeleton, one must consider physical and biological parameters aiming at a better HMI (Zorkot et al., 2022). For this, after the development of the G-Exos, experiments were carried out with volunteers with foot drop, analyzing the following parameters: functionality and usability, accuracy of the onboard system for identifying the gait phases, gait speed, and range of motion of dorsiflexion, eversion/inversion of the ankle, comparing with and without the use of the G-Exos.

### Functionality and usability

Regarding the functionality and usability of the system, 10 volunteers with foot drop reported walking facility with the G-Exos mainly to perform dorsiflexion and ankle stability by controlling the ankle eversion and inversion movements.

Kwon et al. (2019) developed a wearable exoskeleton that showed improvement in both gait propulsion and foot drop prevention with a hemiparetic volunteer (*N* = 1). The G-Exos also improved gait propulsion and foot drop prevention, validated with 10 volunteers with foot drop (in this work, seven with Stroke, two with SCI, and one with ATM). Furthermore, when compared to the work of Kwon et al. (2019), the G-Exos still promotes ankle stabilization through ankle eversion and inversion assistance. Xia et al. (2020) advanced the development of a wearable exoskeleton for foot drop, reporting significant results in ankle eversion/inversion support, but only with healthy volunteers.

It is important to emphasize that the G-Exos was developed with an initial focus on hemiparetic patients, who have motor impairment in only one leg, with the potential to improve neuromuscular coordination (Zhu et al., 2021). On the other hand, the volunteers with SCI have both lower limbs affected. In this context, it was possible to perceive the system to be more functional for the hemiparetic volunteers than for the volunteers with SCI. This can be explained by the fact that the G-Exos promoted gait assistance in only one limb, which may cause gait compensation in the volunteers with SCI (Xue et al., 2022). For having only one leg with sequelae, the G-Exos also showed good functionality for volunteer N10 with acute mielytis. Furthermore, in a general context, the 10 volunteers considered it easier to perform the walk with the G-Exos and would indicate the G-Exos for rehabilitation interventions in patients with foot drop.

Featuring a hybrid system that can be configured for both hybrid, active, and passive modes, the G-Exos weighs < 2 kg and provides ~6 kg of assistance for the ankle dorsiflexion movement. In a general context, the average weight of exoskeletons used for stroke is ~8.9 kg (Rodríguez-Fernández et al., 2021). On a state-of-the-art comparison level, the Restore (ReWalk, USA) weighs ~5 kg (Jasinski, 2020). However, it is important to note that the Restore already has a more robust commercial model, while the G-Exos is a prototype. The battery life, in the hybrid and active system, lasts ~3 h, featuring an autonomy similar to other wearable exoskeleton works developed for foot drop (Kwon et al., 2019; Xia et al., 2020). Regarding the battery, the state-of-the-art exoskeletons developed generally have a battery life of 2–4 h of continuous use (Rodríguez-Fernández et al., 2021). The approximate cost, exclusive of material, for the development of the G-Exos prototype was ~$334.00.

### G-Exos accuracy in identifying gait phase

The identification of gait events is critical for the best performance of the G-Exos, in controlling the active system and providing better HMI. In this context, the gait behavior of each volunteer was also processed and analyzed for comparison with the literature (Chang et al., 2016; Hutabarat et al., 2021). In both cases, it could be seen that the gait phase identification was effective for both the right and left leg, validating the use of the G-Exos for both legs.

Video gait analysis allowed to detect the contact and swing phases by the system for gait phase identification, in which the G-Exos presented an accuracy higher than 94%. Individual analysis showed the lowest accuracy in identifying the gait phase with volunteers N5 and N9. A possible explanation is due to the fact that these volunteers have greater spasticity and ankle inversion, having a short support phase percentage when performing the gait (Zhu et al., 2021) and reducing the accuracy of the reference coordinates of the onboard IMU sensor. Thus, from the results analyzed, it is believed that the system presented lower accuracy with these volunteers due to this gait compensation (Zhu et al., 2021) and, consequently, greater difficulty in detecting the variations of the contact and swing phases.

Analysis of IMU sensors for ankle amplitude in dorsiflexion and plantar flexion movements showed similar curve behavior presented in the literature (Chang et al., 2016; Hutabarat et al., 2021). Furthermore, it can be seen that the ankle shows similar behavior in the hybrid system and the natural gait with foot drop without the G-Exos. This may indicate that the G-Exos allows these patients to perform a gait closer to the natural gait. Furthermore, when compared to the gait with the foot drop, the use of the hybrid system allowed a greater amplitude of the dorsiflexion movement, as can be seen in the indicated valleys that represent the dorsiflexion movement.

### Gait speed

The gait velocity of the volunteers with foot drop was analyzed using the videos recorded with the use of the system, configured in the hybrid system, active and passive, and without the use of the system. The results were compared with the gait without the use of G-Exos to verify whether it promotes a greater speed for the execution of the gait by assisting the ankle dorsiflexion and eversion/inversion movements.

Because of the effort and physical limitations, some of the volunteers did the trial without the G-Exos only before or after. Therefore, it was not possible to analyze whether there was an increase in walking speed with the use of G-Exos for volunteers N2, N4, N7, and N10 As given in Table 4, these volunteers presented an increase in velocity after the use of G-Exos. This may have occurred because the experiment with G-Exos may have promoted muscle stimulation and greater safety for step execution and, consequently, an increase in gait speed, a result that should be further explored in future studies.

In this context, it is believed that the best analysis to be performed is the comparison of the use of G-Exos (hybrid, active and passive) with walking without the use of it before. Thus, in a general context, we given in Table 4, the increase in gait speed with the use of G-Exos, when compared to “Before G-Exos,” in all the volunteers analyzed. Volunteers N1, N3, N5, and N6 performed the gait without G-Exos at the beginning (Variable “Before G-Exos” in Table 4) and end of the experiment and it is possible to observe a temporary increase in speed, which could possibly be explained by muscle stimulation and greater safety for step execution.

When compared with wearable exoskeletons in the literature, it is possible to realize that the existing active systems also help to increase the gait speed (Awad et al., 2017). With the G-Exos, we also analyzed the increase in gait speed through the passive system mechanism that makes use of the natural human biomechanics, in which good results were obtained that should be further explored.

It is worth mentioning that this analysis was performed by custom Matlab scripts developed by the authors and by the analysis of the records of the experiment, in which some variables may affect the accuracy, such as displacement performed by the volunteers, and variations in the experimental design due to the limitations of the volunteers with foot drop.

Even presenting relevant results with a small sample size, we aim to confirm the hypothesis that the natural gait velocity increases significantly after the use of the G-Exos with further experiments with larger and more homogeneous groups. Furthermore, a statistical analysis of the increase in gait speed will be carried out with the data collected.

### Statistical analysis

In the statistical analysis, in a general context, the G-Exos presented a significant difference in the increase of the amplitude of dorsiflexion movement and reduction of the amplitude in the ankle eversion and inversion movements, important for promoting gait and ankle stability (Galang, 2019), when compared with the gait without the use of G-Exos on the ground. In this case, the three configurations tested on the G-Exos were shown to be effective in assisting dorsiflexion and ankle eversion and inversion.

#### Ankle dorsiflexion range of motion

In the ankle dorsiflexion movement during gait, an increase in range of motion was expected to occur with the use of G-Exos in the hybrid active and passive system when compared to gait without the use of G-Exos. In the following statistical analyses, we check whether this actually occurred.

For the foot drop group, there is evidence of a difference in the increase in ankle range of motion with the use of G-Exos in the volunteers, for the hybrid, active and passive systems, when compared with the gait samples without the use of G-Exos. It can also be seen that there was a significant difference in the performance of the dorsiflexion movement on the ground and between the hybrid and active and hybrid and passive systems.

#### Range of motion of ankle inversion and eversion

In the inversion/eversion movement of the ankle during gait, a decrease in range of motion was expected with the use of G-Exos when compared to gait without the use of G-Exos. This result was expected mainly in the hybrid and passive systems because they have elastic bandages, parallel to the ankle, that allow the control of eversion and inversion movement.

There is evidence of a difference in the decreased amplitude of ankle eversion and inversion movement with the use of G-Exos in the volunteers with foot drop, for the hybrid, active and passive systems, when compared with the gait samples without the use of G-Exos. It is also possible to verify that there was a significant difference in performing the eversion and inversion movements on the ground l between the hybrid and active and passive systems.

In the individual statistical analysis, there is evidence of a difference in the decrease in amplitude of ankle eversion and inversion movements with the use of G-Exos, for the hybrid, active and passive systems, when compared to the gait samples without the use of G-Exos on the ground. It is worth noting that there is no evidence of a difference in the decreased amplitude of ankle eversion and inversion movements with the use of G-Exos for the N3 and N4 volunteers. The volunteer N3 presents significant ankle stability, controlling the ankle eversion and inversion movements. This fact may show that there was no need for the G-Exos assistance.

In the analysis of volunteer N4, it was possible to verify that due to the high spasticity presented and ankle eversion/inversion, the G-Exos was not effective in promoting assistance in these specific movements. Thus, future studies should analyze the G-Exos functionality according to the degree of spasticity of the volunteers.

In a general context, it was possible to obtain statistical validation and prove that the G-Exos assisted the gait of the volunteers with foot drop by assisting the ankle dorsiflexion, inversion, and eversion movements. The three configurations tested in the G-Exos were effective in assisting the aforementioned movements.

Even though the results showed significant assistance in gait, the experiment was carried out with a small sample size and needs a larger and more homogeneous sample group. We believe that G-Exos could present a greater usability for the development of day-to-day tasks, and it could possibly be used beyond the clinical application helping the human being in alleviating the symptoms of different diseases.

The G-exos system, composed of an active and passive system, allows individuals with gait impairment to improve their walking abilities helping them to raise their foot and also in the ankle stability. We believe that the G-Exos could be successfully used intensively for walk rehabilitation for different conditions in clinics, rehabilitation centers and hospitals, as well as be used for daily life activities promoting independence for patients, caregivers, and their families. This will allow a fully social and accessible inclusion of these patients (Meneses et al., 2019).

## Data availability statement

The raw data supporting the conclusions of this article will be made available by the authors, without undue reservation.

## Ethics statement

The studies involving human participants were reviewed and approved by Ethics Committee of the Santos Dumont Institute, CAAE: 41184020.4.0000.0129. The patients/participants provided their written informed consent to participate in this study.

## Author contributions

MZ: developing G-Exos, running the experiment, data processing and analyzing results, and writing. LD: supporting the development of G-Exos, support of the experiment, and data processing. EM: guidance and support to the development of G-Exos, support, monitoring of the experiment, and writing review. FB: guidance and support to the development of G-Exos, analysis of results, and writing review. All authors contributed to the article and approved the submitted version.

## Funding

This work was supported by Technological Development (CNPq) from the Brazilian government (number 428699/2016-2) and to the Graduate Program in Neuroengineering, Edmond and Lily Safra International Institute of Neuroscience, Santos Dumont Institute.

## Conflict of interest

The authors declare that the research was conducted in the absence of any commercial or financial relationships that could be construed as a potential conflict of interest.

## Publisher's note

All claims expressed in this article are solely those of the authors and do not necessarily represent those of their affiliated organizations, or those of the publisher, the editors and the reviewers. Any product that may be evaluated in this article, or claim that may be made by its manufacturer, is not guaranteed or endorsed by the publisher.
