# Corrigendum: G-Exos: A wearable gait exoskeleton for walk assistance

**DOI:** 10.3389/fnbot.2022.1098880

**Published:** 2022-12-01

**Authors:** Mouhamed Zorkot, Léa Ho Dac, Edgard Morya, Fabrício Lima Brasil

**Affiliations:** ^1^Neuroengineering Program, Edmond and Lily Safra International Institute of Neuroscience, Santos Dumont Institute, Macaiba, Brazil; ^2^Swiss Federal Institute of Technology, School of Life Sciences, Lausanne, Switzerland

**Keywords:** exoskeleton, foot drop, gait, assistive technology, human–machine interaction

In the published article, there was an error in Figure 1 as published. For safety, the Figure 1 should be modified. The corrected Figure 1 and its caption appear below. The picture is of the authors' own authorship. Thus, the reference Palastanga et al. should be removed.

**Figure 1:**
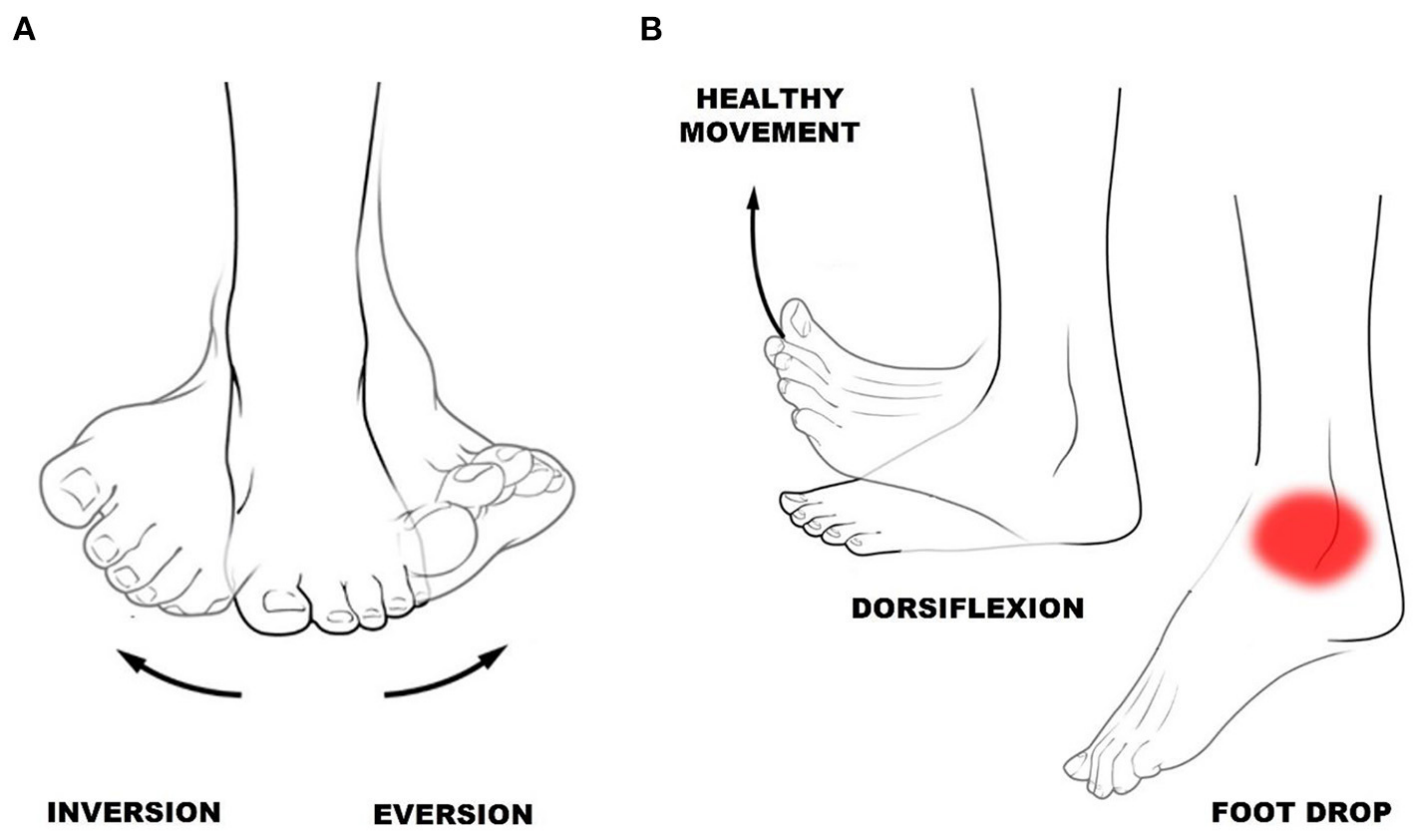
**(A)** Ankle inversion and eversion. **(B)** Ankle dorsiflexion—toes toward shins, and foot drop—difficulty lifting the forefoot.

In the published article, the reference for Palastanga and Soames (2011) [Palastanga, N., and Soames, R. (2011). *Anatomy and Human Movement, Structure and Function with PAGEBURST Access, 6: Anatomy and Human Movement*. Amsterdam: Elsevier Health Sciences] will not be used anymore as the figure in the original article was adapted from this paper. It should be removed.

The authors apologize for this error and state that this does not change the scientific conclusions of the article in any way. The original article has been updated.

## Publisher's note

All claims expressed in this article are solely those of the authors and do not necessarily represent those of their affiliated organizations, or those of the publisher, the editors and the reviewers. Any product that may be evaluated in this article, or claim that may be made by its manufacturer, is not guaranteed or endorsed by the publisher.

