# Supplementary figures and images for: G-Exos: A wearable gait exoskeleton for walk assistance
Source: Front Neurorobot. 2022 Nov 10;16:939241. doi: 10.3389/fnbot.2022.939241 (PMC9684314; doi:10.3389/fnbot.2022.939241)

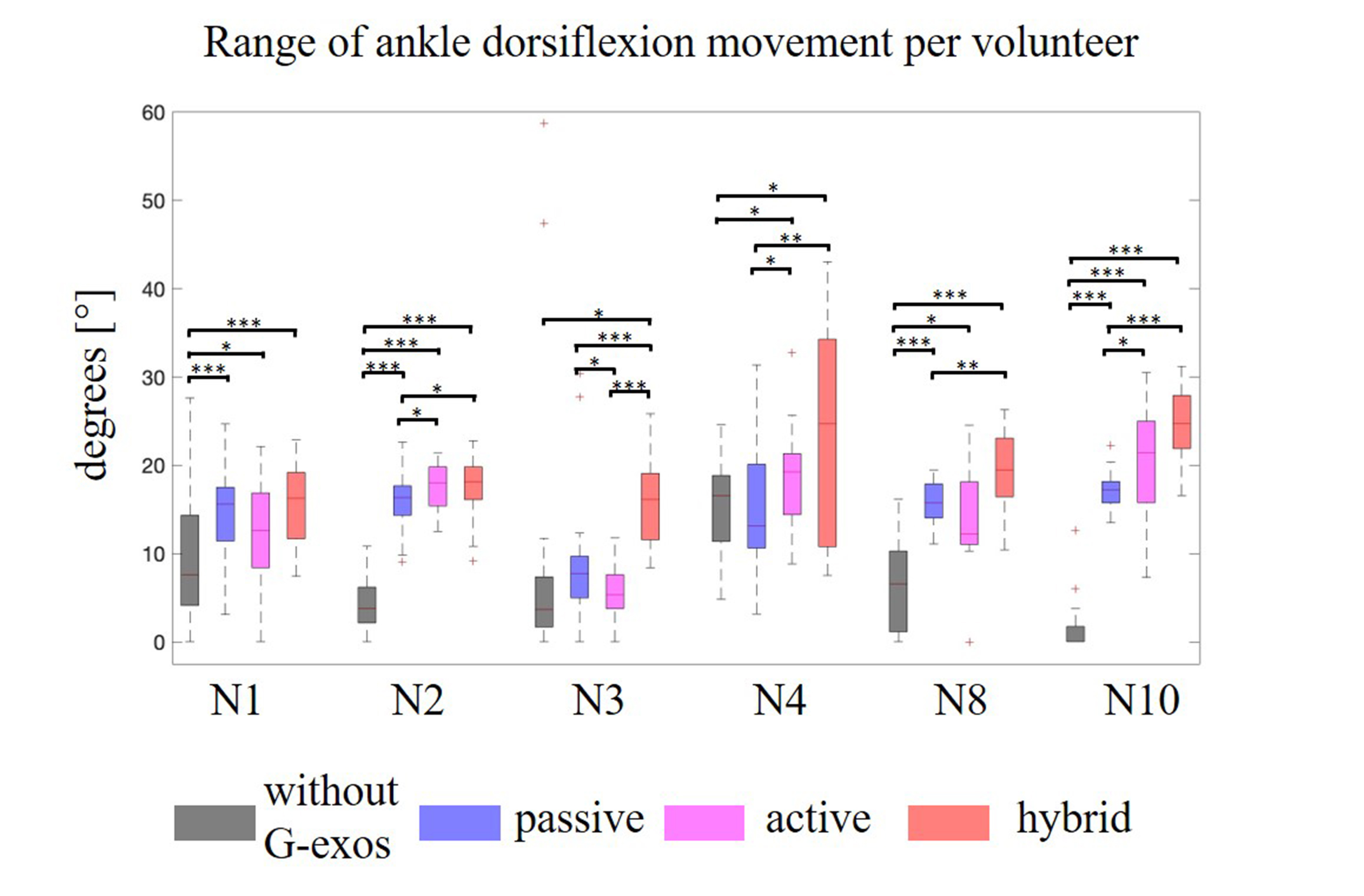

Supplement: Supplementary file 1 [file Image_1.JPEG]

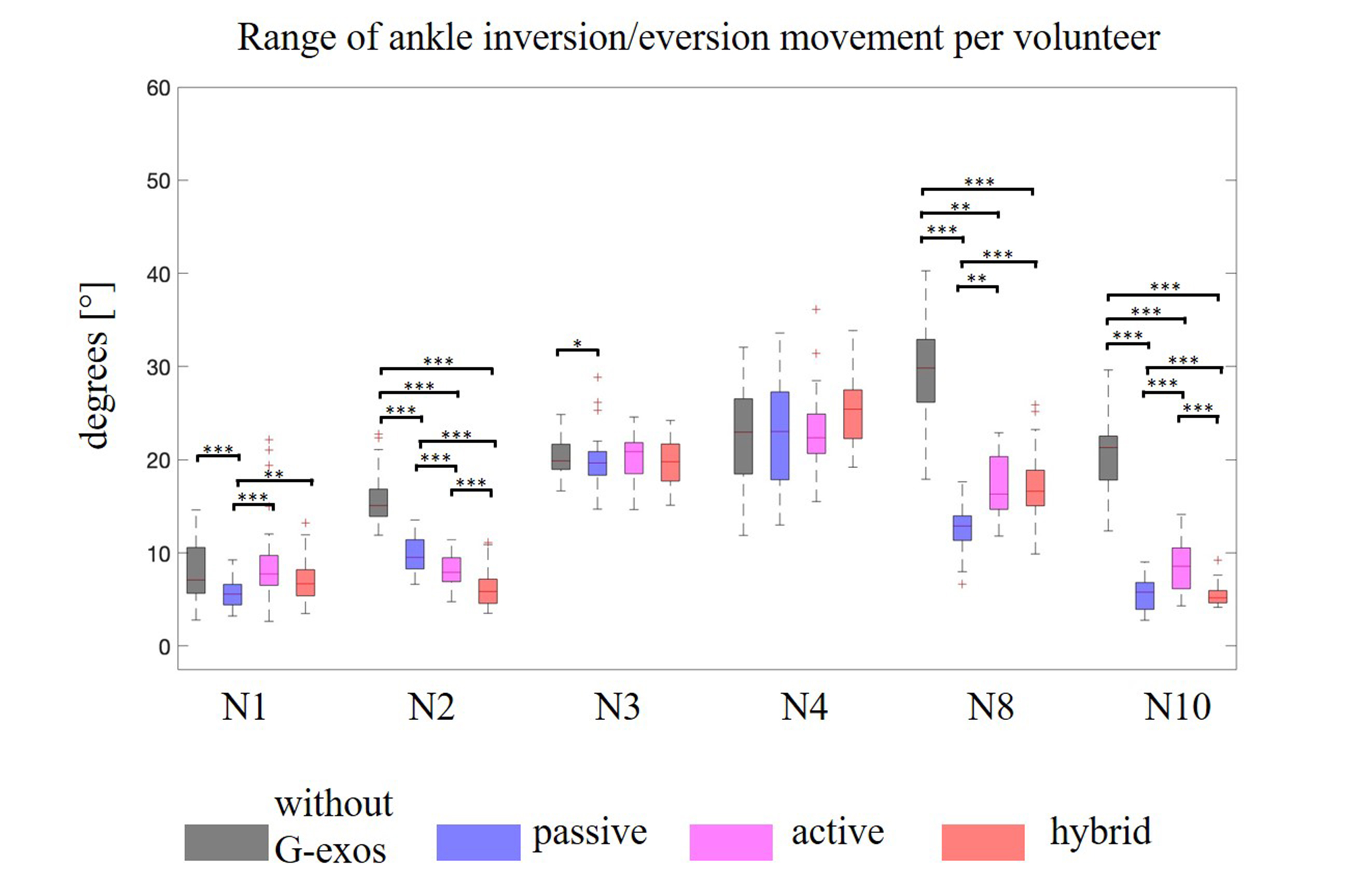

Supplement: Supplementary file 2 [file Image_2.JPEG]
